# Bloodstream bacterial infection among outpatient children with acute febrile illness in north-eastern Tanzania

**DOI:** 10.1186/s13104-015-1178-9

**Published:** 2015-07-03

**Authors:** Coline Mahende, Billy Ngasala, John Lusingu, Allvan Butichi, Paminus Lushino, Martha Lemnge, Bruno Mmbando, Zul Premji

**Affiliations:** Korogwe Research Laboratory, Tanga Centre, National Institute for Medical Research, P. O. Box 5004, Tanga, Tanzania; Department of Medical Entomology and Parasitology, School of Public Health, Muhimbili University of Health and Allied Sciences, P. O. Box 65001, Dar es Salaam, Tanzania; Department of International Health, Microbiology and Immunology, University of Copenhagen, Copenhagen, Denmark

**Keywords:** Bacterial infection, *Salmonella typhi*, Malaria, Children, Antimicrobial sensitivity, Korogwe, Tanzania

## Abstract

**Background:**

Fever is a common clinical symptom in children attending hospital outpatient clinics in rural Tanzania, yet there is still a paucity of data on the burden of bloodstream bacterial infection among these patients.

**Methods:**

The present study was conducted at Korogwe District Hospital in north-eastern Tanzania. Patients aged between 2 and 59 months with a history of fever or measured axillary temperature ≥37.5°C attending the outpatient clinic were screened for enrolment into the study. Blood culturing was performed using the BACTEC 9050® system. A biochemical analytical profile index and serological tests were used for identification and confirmation of bacterial isolates. In-vitro antimicrobial susceptibility testing was performed using the Kirby-Bauer disc diffusion method. The identification of *Plasmodium falciparum* malaria was performed by microscopy with Giemsa stained blood films.

**Results:**

A total of 808 blood cultures were collected between January and October 2013. Bacterial growth was observed in 62/808 (7.7%) of the cultured samples. Pathogenic bacteria were identified in 26/808 (3.2%) cultures and the remaining 36/62 (58.1%) were classified as contaminants. *Salmonella typhi* was the predominant bacterial isolate detected in 17/26 (65.4%) patients of which 16/17 (94.1%) were from patients above 12 months of age. *Streptococcus pneumoniae* was the second leading bacterial isolate detected in 4/26 (15.4%) patients. A high proportion of *S.typhi* 11/17 (64.7%) was isolated during the rainy season. *S. typhi* isolates were susceptible to ciprofloxacin (n = 17/17, 100%) and ceftriaxone (n = 13/17, 76.5%) but resistant to chloramphenicol (n = 15/17, 88.2%). *P. falciparum* malaria was identified in 69/808 (8.5%) patients, none of whom had bacterial infection.

**Conclusion:**

Bloodstream bacterial infection was not found to be a common cause of fever in outpatient children; and *S. typhi* was the predominant isolate. This study highlights the need for rational use of antimicrobial prescription in febrile paediatric outpatients presenting at healthcare facilities in rural Tanzania.

## Background

Bacterial bloodstream infections are known common causes of febrile illnesses especially among children living in resource poor areas of sub-Saharan Africa [1]. The epidemiology of bacterial infections varies across the world depending on geographical environment, social-economical status and the underlying illnesses such as malaria and human immunodeficiency virus (HIV) [2–4]. Invasive bacterial infections among hospitalized children have been extensively studied yet limited data exist on the disease burden among outpatient children living in rural settings of Tanzania [5].

Bacterial infections among inpatient children in many parts of sub-Saharan Africa have been documented to be caused mainly by non-typhoidal *Salmonella*, *Streptococcus pneumoniae*, *Haemophilus influenzae* and *Escherichia coli* [1, 6, 7]. Current data indicate differing and changing pattern of bacterial infections in areas with declining malaria and HIV transmissions [3, 8–11]. Therefore, understanding the local epidemiology of pathogens is essential for early disease interventions.

The general clinical symptoms observed in patients with invasive bacterial infections include fever, headache, abdominal pain, vomiting, diarrhoea and malaise [12]. Like other febrile illnesses, these signs and symptoms are non-specific and may overlap with other febrile infections making diagnosis in children difficult and challenging in the absence of appropriate laboratory confirmation [13, 14]. The majority of healthcare facilities with the exception of a few research laboratories in rural African countries, lack adequate microbiologic blood culture facilities; which is the universally preferred and effective standard method for detection of bacterial pathogens [15]. As a result, clinicians incorrectly diagnose and treat patients based on clinical symptoms rather than laboratory evidence. Consequently, patients are often given the wrong diagnosis, which leads to inappropriate treatment, poor prognosis and promotion of antimicrobial drug resistance [16, 17].

Empirical fever treatment with antimicrobials such as amoxicillin and chloramphenicol obtained over the counter without prescription is common in Tanzania, and this accentuates the problem of antimicrobial resistance [18]. Multiple drug resistance among Gram negative bacterial isolates has been spreading and increasing globally over the years [19–21]. The antimicrobial susceptibility patterns vary with geographical location, therefore, periodic surveillance of local patterns is crucial in monitoring this problem as well as for correct prescriptions and future targets for treatments.

A majority of studies have documented bacterial infections among hospitalised patients who are usually severely ill and already exposed to antimicrobial drugs before seeking professional medical care [22]. In Tanzania, data on the burden of bacterial infection among children attending outpatient clinics in rural hospitals is lacking. With the current observations on changing patterns of bacterial infections, local prevalence and antimicrobial resistance profile need to be characterised for timely interventions. The objective of the study was to identify bloodstream bacterial infections causing fever in young children attending outpatient clinic at Korogwe District Hospital in north-eastern Tanzania. The study characterised the in vitro antimicrobial susceptibility pattern of bacterial isolates and provided an outline on the clinical characteristics of patients with bacterial infections.

## Methods

### Study area

**Figure 1:**
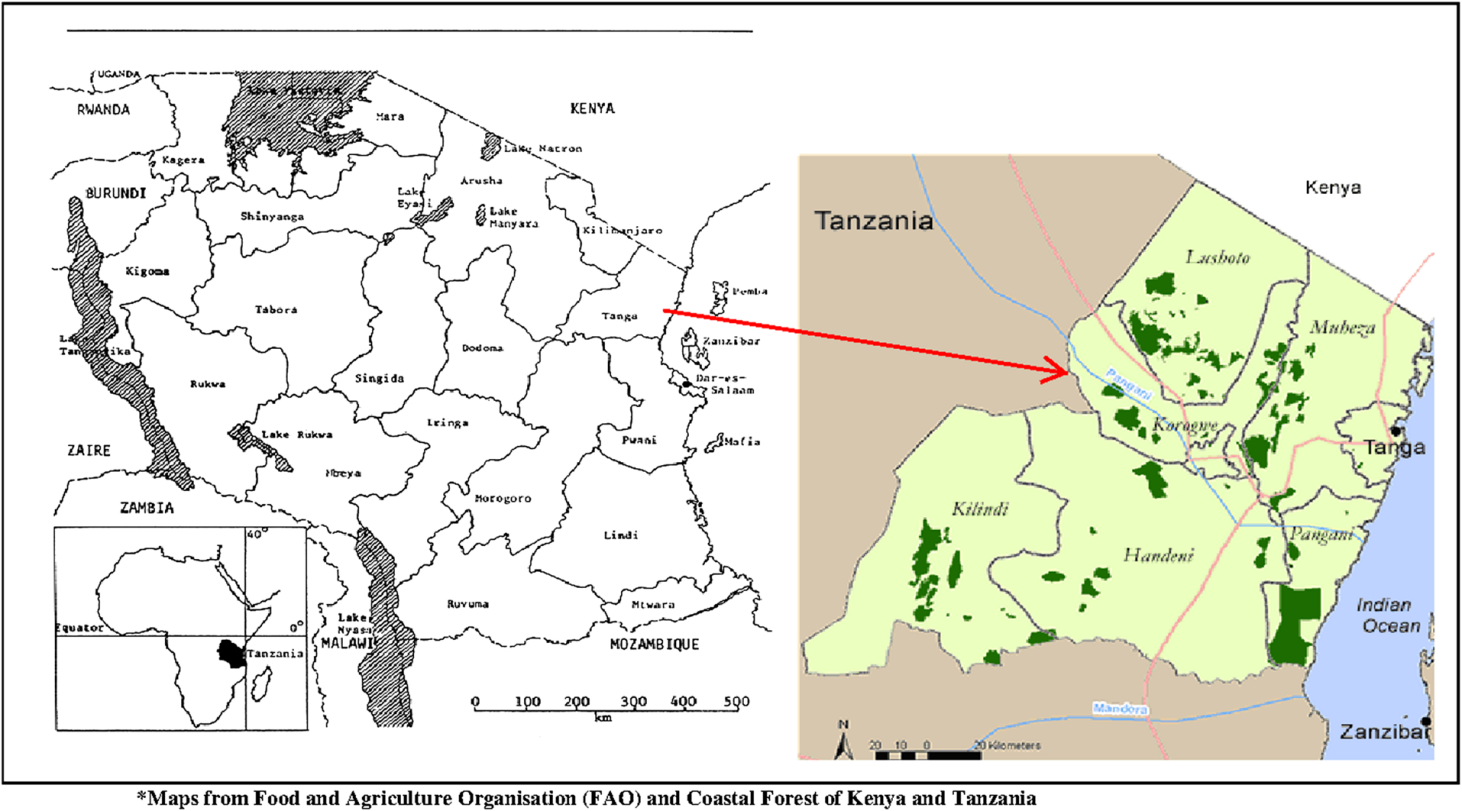
A map showing study area.

The study was conducted at Korogwe District Hospital (KDH) in Korogwe District north-eastern Tanzania (Figure 1). Approximately 73,275 children under the age of 5 years live in Korogwe District [23]. The District experiences temperatures between 18 and 20°C during the rainy season and 26–30°C during the dry season. The annual rainfall ranges from 700 to 1,000 mm with long rainy seasons extending from March to May and short rains in September and October. The majority of inhabitants (80%) reside in rural settings, practicing subsistence farming and informal trade. The hospital receives around 6,000 (2012 estimates) outpatient visits from under fives annually. The Korogwe District is an area with declining malaria transmission with a prevalence of around 13% according to surveys conducted from 2003 to 2008 [24]. Pneumococcal and rotavirus vaccines were introduced in Tanzania as part of the Expanded Program on Immunization (EPI) in January 2013. The administrative coverage for other vaccines in the EPI has been above 90% (Korogwe Primary Health Care report, District Medical Officer, personal communication). These vaccines include; Bacille Calmette–Guérin, pentavalent vaccine (diphtheria–tetanus–pertussis, hepatitis B and haemophilus influenza type B), poliovirus and measles vaccines.

### Study participants

The study was conducted from January 2013 to October 2013 where febrile children presenting at KDH outpatient clinic were assessed for enrolment. The inclusion criteria were: children aged between 2 and 59 months presenting at KDH with a history of fever in the last 48 h or measured axillary temperature of ≥37.5°C at presentation. Exclusion criteria included previous consultation for the presenting complaints, intake of antimalarial and/or antibiotic drugs within the last 7 days, planned admissions (e.g. elective surgery) and trauma/injury.

### Ethics statement

The study was granted ethical clearance with reference number NIMR/HQ/R.8a/Vol.1X/1373 from the Tanzanian Medical Research Coordinating Committee. Parent/legally accepted guardian of every child enrolled in the study provided a written informed consent by either signature or thumb print for the illiterate parent/legally accepted guardian.

### Study procedures

A detailed medical history and a thorough clinical examination were performed on each patient and information was entered into a standardised case record form. This included demographic information, clinical history, physical examination data, vital signs (axillary temperature and respiratory rate) and body weight. Clinical diagnosis was made based on presenting signs and symptoms according to Integrated Management of Childhood Illness (IMCI) guidelines [25] so that patients could be managed appropriately according to the national standard practice [26, 27] while waiting for blood culture results.

### Blood sampling and laboratory investigations

A maximum of 5 ml of venous blood was collected for laboratory investigations using aseptic techniques. Blood was drawn after disinfecting the venipuncture site with 70% isopropyl alcohol and iodine. A blood volume of between 1 and 3 ml was inoculated into commercial BD BACTEC PEDS PLUS^®^ culture bottles. A single aerobic culture bottle was used per patient. The inoculated blood culture bottles were immediately (that was within 2 h) incubated at 35°C in the BACTEC 9050^®^ (Becton–Dickinson, Sparks, MD, USA) culture system for a maximum of 5 days unless flagged positive. Sheep-blood, MacConkey and chocolate agar plates with the use of routine microbiological techniques were used for sub-culturing positive samples according to standard methods. Analytical Profile Index (API) biochemical test kit (BioMérieux^®^™, France) was used to identify pathogens. *Salmonella* isolates were confirmed by serological tests using specific antisera (Becton–Dickinson, Sparks, MD, USA).

In-vitro antimicrobial susceptibility testing for pathogenic bacterial isolates was performed using the Kirby-Bauer disc diffusion method. The antimicrobial agents tested included; amoxicillin (10 µg), cefoxitin (30 µg), ceftriaxone (30 µg), chloramphenicol (30 µg), ciprofloxacin (5 µg), clarithromycin (2 µg), co-trimoxazole (1.25 µg), gentamicin (10 µg), penicillin (1 µg) and tetracycline (30 µg). Clinical and Laboratory Standards Institute (CLSI) criteria were used in the interpretation of zone sizes [28].

The identification of malaria infection was performed by microscopy. Thick and thin blood smears were prepared from venous blood collected in the ethylene diamine tetra acetic acid (EDTA) tubes. Blood slides were stained with a 5% Giemsa solution for 30 min and examined for asexual *Plasmodium falciparum* parasites. The parasite densities were calculated against 200 (500 if parasite count was <10) leucocytes multiplied by the patient’s leucocyte count and expressed as parasites/μl. At least 100 high power microscopic fields of the thin film were examined to exclude the diagnosis of malaria. The slides were independently double-read by experienced microscopists and in case of discrepancy, a third reading was performed. The complete blood count was analysed using the automated MS4s haematological analyser (Melet Schloesing Diamond Diagnostics, USA).

### Quality control and assessment

Laboratory investigations were carried out at Korogwe Research Laboratory of the Tanzanian National Institute for Medical Research. Quality control was performed for all laboratory methods according to standard guidelines [29]. During the study, the laboratory participated in the bacteriology external quality assessment programme with the National Institute for Communicable Diseases (NICD) registration number 107.

### Case definitions

Fever was defined as history of abnormally high body temperature reported by the parent/guardian or measured axillary temperature ≥37.5°C on presentation. Bloodstream bacterial infection (bacteraemia) was defined as fever with isolation of pathogenic bacteria from a positive blood culture. A contaminant was defined as non-pathogenic bacteria (mostly from skin) such as coagulase-negative *Staphylococcus* species, *Corynebacterium* species, *alpha*- *or gamma*-*hemolytic streptococci, Micrococcus* species*, Bacillus* species and *Propionibacterium* species. However, a definitive diagnosis of a contaminant was decided by a clinician based on the clinical presentation of the patient considering that these organisms could be the cause of an opportunistic infection. The monthly cut-off point for contamination rate during the study was 6%. Malaria infection was defined as fever with the presence of asexual *P. falciparum* parasites in a blood smear confirmed by microscopy. Clinical diagnoses were defined according to the IMCI guidelines [25]. Anaemia was defined as haemoglobin concentration below 9.3 g/dL. The reference interval used was 4.5–17 × 10^3^/µL for leucocyte count.

### Data management and statistical analysis

Data management involved double entry and validation using Microsoft Access 2007. Data analysis was done by STATA version 11.2 (Stata Corp LP, College Station, TX, USA). Variables were summarized as frequencies and percentages, medians and inter-quartile ranges as appropriate. Categorical data were compared by the Chi-square test. The logistic regression (odds ratio and 95% confidence interval) was used to determine association of clinical diagnosis at enrolment with the occurrence of bacteraemia. The *P* value <0.05 was considered significant.

## Results

During the study period of January 2013 to October 2013, a total of 1,380 patients were screened for enrolment. Five hundred and thirteen patients (37.2%) did not meet the inclusion criteria; 51/513 (9.9%) patients were not within the required age group, 153/513 (29.8%) patients were afebrile, 219/513 (42.7%) patients had used antimalaria/antimicrobial drug within 7 days prior to screening and 90/513 (17.5%) patients refused participation. The remaining 867/1,380 (62.8%) patients were recruited of whom 808 had blood culture samples collected.

The median age of patients was 15.6 months (Inter-quartile range: 8.9–30.0). The sex ratio was 431/808 (53.3%) boys and 377/808 (46.7%) girls. The demographic profile of patients is indicated in Table 1.

**Table 1:** Demographic characteristics and clinical diagnosis of patients with bacteraemia

	Bacteraemia positive (n = 26)	Bacteraemia negative (n = 782)	*P* value
Girls	11 (42.3)	366 (46.8)	0.65
Age			
2–35 months	20 (76.9)	654 (83.6)	0.40
36–59 months	6 (23.1)	128 (16.4)	
Axillary temperature (°C), median (IQR)	38.3 (37.6–39.3)	38 (37.5–38.7)	
Axillary temperature ≥39°C	7 (26.9)	116 (14.8)	0.09
Symptoms			
Cough	5 (19.2)	360 (46.0)	0.01
Shortness of breath	2 (7.7)	87 (11.1)	0.58
Diarrhoea	6 (23.1)	155 (19.8)	0.68
Clinical diagnosis at enrolment			
Malaria	0 (0)	69 (8.8)	0.11
Gastroenteritis	8 (30.8)	151 (19.3)	0.15
Upper respiratory tract infection	3 (11.5)	309 (39.5)	<0.01
Pneumonia	2 (7.7)	114 (14.6)	0.33
Other infections^a^	1 (3.8)	59 (7.5)	0.17
Multiple infections^a^	3 (11.5)	140 (17.9)	0.40
Non-specific febrile illness	12 (46.2)	80 (10.2)	<0.01
Laboratory findings (n = 785)			
Anaemia	6/26 (23.1)	203/759 (26.7)	0.68
Leucocyte count (10^3^/µL), median (IQR)	10.4 (7.5–16.3)	11.5 (8.6–15.7)	
Leucocyte count >17 (10^3^/µL)	6/26 (23.1)	157/759 (20.7)	0.77

Data are n (%), IQR inter-quartile range.

^a^Conjunctivitis, fungal infection, gingivitis, hookworm infection, otitis media, skin infection, urinary tract infection.

Bacterial growth was observed in 62/808 (7.7%) of the cultured samples. Single pathogenic bacteria were isolated from 26/808 (3.2%) cultures. The remaining 36/62 (58.1%) were classified as contaminants. Coagulase-negative staphylococcus was the most frequently isolated contaminant with 21/36 (58.3%) of the bacterial isolates followed by micrococcus 9/36 (25%), *Corynebacterium species* 3/36 (8.3%), lactobacillus 1/36 (2.8%), *Sphingomonas paucimobilis* 1/36 (2.8%) and *Citrobacter braakii* 1/36 (2.8%). Blood cultures that flagged positive from the BACTEC 9050^®^ culture system with no bacteria growth accounted for 8/808 (0.9%) of the positive cultures.

Gram negative bacteria were the leading pathogenic bacterial isolates compared to the Gram positive bacteria. *Salmonella typhi* was the most prevalent bacterial isolate identified in 17/26 (65.4%) cultures followed by *S. pneumoniae* at 4/26 (15.4%) and others as shown in Table 2. Pathogenic bacteria (n = 20/26, 76.9%) were commonly isolated from patients below 36 months of age. The majority of *S. typhi* 16/17 (94.1%) were isolated from patients above 12 months of age whilst non-typhoidal *Salmonella* 2/2 (100%) were from patients below 12 months of age (Table 2). A high proportion of *S.typhi*, 11/17 (64.7%), was isolated during the rainy season (March to May and September to October). Bloodstream bacterial infection was not associated with gender (*χ*^2^ = 0.2, *P* = 0.65).

**Table 2:** Distribution of pathogenic bacterial isolates (%) according to age groups

	Age group			
	2–11 months	12–35 months	36–59 months	Total n (%)
Blood cultures collected	320	354	134	808
Gram positive bacteria				
*Staphylococcus aureus*	0 (0)	1 (6.7)	0 (0)	1 (3.8)
*Streptococcus pneumoniae*	1 (20)	3 (20)	0 (0)	4 (15.4)
Gram negative bacteria				
*Enterobacter cloaccae*	0 (0)	1 (6.7)	0 (0)	1 (3.8)
*Escherichia coli*	1 (20)	0 (0)	0 (0)	1 (3.8)
Non-typhoidal *Salmonella*	2 (40)	0 (0)	0 (0)	2 (7.7)
*Salmonella typhi*	1 (20)	10 (66.7)	6 (100)	17 (65.4)
Total bacterial isolates	5	15	6	26

Clinical diagnosis and laboratory findings at enrolment of patients with bacteraemia were compared with those without bacteraemia (Table 1). The proportion of bacteraemic patients with measured axillary temperature of ≥39°C was not of statistical significance (*χ*^2^ = 2.85, *P* = 0.09) when compared to the non-bacteraemic group. Out of 159/808 (19.7%) patients with clinical diagnosis of gastroenteritis, 8/159 (5%) patients had positive blood cultures and among them, six were *S. typhi* isolates. One *S. pneumoniae* isolate was identified from a patient with a clinical diagnosis of pneumonia. *P. falciparum* malaria was identified in 69/808 (8.5%) patients, none of whom had bacterial infection. Patients with the clinical diagnosis of non-specific febrile illness were at higher risk of having bacteraemia (OR = 7.6, 95% CI 3.3–17.4), *P* value <0.01). Eight *S. typhi* and two *S. pneumoniae* isolates were identified from these patients.

In-vitro antimicrobial susceptibility testing to selected antimicrobial agents was assessed for all pathogenic bacterial isolates. *S. typhi* isolates were susceptible to ciprofloxacin 17/17(100%) and resistant to chloramphenicol 15/17 (88.2%). A susceptibility rate of 100% was observed for *S. pneumoniae* isolates to chloramphenicol. All bacterial isolates were observed to have high rates of resistance to amoxicillin and co-trimoxazole (Table 3).

**Table 3:** *In*-*vitro* antimicrobial susceptibility pattern of bacterial isolates

Antimicrobial agent	Susceptible n (%)	Intermediate n (%)	Resistant n (%)
*Salmonella typhi* (n = 17)			
Amoxicillin	2 (11.8)	0 (0)	15 (88.2)
Ceftriaxone	13 (76.5)	3 (17.6)	1 (5.9)
Chloramphenical	2 (11.8)	0 (0)	15 (88.2)
Ciprofloxacin	17 (100)	0 (0)	0 (0)
Gentamicin	8 (47.1)	5 (29.4)	4 (23.5)
Co-trimoxazole	1 (5.9)	2 (11.8)	14 (82.4)
*Streptococcus pneumoniae* (n = 4)			
Amoxicillin	1 (25)	0 (0)	3 (75)
Cefoxitin	2 (50)	1 (25)	1 (25)
Ceftriaxone	3 (75)	1 (25)	0 (0)
Chloramphenicol	4 (100)	0 (0)	0 (0)
Clarithromycin	3 (75)	0 (0)	1 (25)
Co-trimoxazole	0 (0)	0 (0)	4 (100)
Penicillin	0 (0)	0 (0)	4 (100)
Other *Enterobacteriaceae* (n = 4)			
Amoxicillin	2 (50)	0 (0)	2 (50)
Ceftriaxone	4 (100)	0 (0)	0 (0)
Chloramphenicol	3 (75)	0 (0)	1 (25)
Ciprofloxacin	4 (100)	0 (0)	0 (0)
Gentamicin	1 (25)	1 (25)	2 (50)
Co-trimoxazole	2 (50)	0 (0)	2 (50)
*Staphylococcus aureus* (n = 1)			
Cefoxitin	0 (0)	0 (0)	1 (100)
Chloramphenicol	1 (100)	0 (0)	0 (0)
Gentamicin	1 (100)	0 (0)	0 (0)
Penicillin	0 (0)	0 (0)	1 (100)

## Discussion

In the current study, bloodstream bacterial infection was not found to be a common cause of fever in outpatient children from rural Tanzania. Three previous studies which investigated bacterial infection similar to the present study also showed a low prevalence of bacteraemia in the range of 1 and 5% [5, 30, 31]. A recent published finding of D’Acremont et al., indicated evidence of a viral origin (70%) to be more common than evidence of a bacterial or parasitic origin in outpatient febrile children from Tanzania [5]. When the distribution of pathogenic bacteria was characterized according to age group, patients below 36 months of age had a higher prevalence of bacterial infection. A significant number of studies on bacteraemia in African children have reported higher prevalence of bacterial isolates from children below 36 months of age similar to what is reported in this paper [20, 32]. Young children are usually susceptible to infections because of their immature immune system [33]. This study did not find any association between gender and bacterial infection in contrast to what was reported by Were and colleagues on the study among Kenyan children [13].

The Gram negative bacteria were the leading isolates with *S. typhi* being the most predominant. This indicates the occurrence of typhoid fever among young pre-school children in this particular community, which was otherwise thought to be uncommon within this age group [7]. Recent published work from sub-Saharan African countries with declining malaria transmissions has reported the predominance of *S. typhi* bacteria over other bacterial isolates (especially the non-typhoidal *Salmonella*) [31, 34, 35]. The current findings from the study which was conducted in an area with low prevalence of malaria infection are in keeping with previous findings [11, 36]. The observed change in the epidemiological pattern of bacterial infections may have been due to the scaling up of malaria interventions, better detection methods of malaria and the introduction of pneumococcal vaccination [37]. Continuous surveillance of bacterial infections is crucial for both current and future disease interventions.

Nearly two-thirds of *S. typhi* isolates were identified during the rainy season suggesting that the environmental risk factors are important to the occurrence of typhoid [38]. Similar associations have been found in Malawi and Sierra Leone where the frequency of *Salmonella* infections has been associated with the rainy season [39, 40]. Therefore, Tanzania and other low-income countries should take measures and work towards protecting water sources in rural areas for control against spreading of the bacteria. This can be attained through good sanitation to safe water and public health education on personal and food hygiene. Simultaneously, researchers and pharmaceutical companies should expedite development of vaccine against typhoid for immunization in children. Currently, typhoid vaccine is available for adult travelers from developed countries [41, 42].

In those patients who had the clinical diagnosis of non-specific febrile illness at enrolment, the use of clinical symptoms and IMCI guidelines missed nearly half of the patients with culture-confirmed enteric fever. Apart from being febrile, these patients did not have other clinical symptoms or an elevated leucocyte count. Such patients would return at a future date with severe conditions requiring inpatient hospital stay. The availability of blood culture facility in the study has highlighted the importance of confirming the diagnosis of febrile illnesses caused by bacterial infections in children seeking hospital care. However, this is a difficult challenge to implement in hospital facilities in resource poor areas. These findings emphasize the need to develop highly sensitive and affordable point of care rapid diagnostic tests for use in resource limited areas to provide prompt detection of bacterial infections and timely initiation of effective antimicrobial therapy. This iterates the need of improving and having adequate microbiology laboratories at secondary healthcare facilities for local surveillance of bacteria pathogens as well as antimicrobial susceptibility patterns.

Chloramphenicol was not effective during the in vitro antimicrobial susceptibility testing of *S.typhi* isolates. Despite previous reports on its poor performance, it has been the recommended first-line antimicrobial of choice to young patients suspected of enteric infections attending healthcare facilities in Tanzania [17, 27]. Meanwhile, chloramphenicol was found to have 100% effectiveness against the Gram positive bacterial isolates. Ceftriaxone and ciprofloxacin were effective antimicrobials for Gram negative bacterial isolates. Ceftriaxone is available for children under 5 years of age, but usually expensive and often administered for inpatients where as ciprofloxacin is not recommended for use in young children due to its adverse effects [27, 43]. This study found low susceptibility of *S. pneumoniae* isolates to amoxicillin despite this drug being commonly used for treating acute respiratory infections in Tanzanian children [27]. These data highlight the need to conduct further studies so as to facilitate policy makers to revise antimicrobial use guidelines accordingly. Periodic in vitro susceptibility testing of antimicrobials is essential to provide guidance for appropriate and effective treatment against bacterial infections as well as understanding the local antimicrobial resistance patterns. Hence, adequate microbiology laboratory facilities are essential and highly needed.

The study had some potential limitations. First, there was no confirmation evidence of prior use of antimicrobial drugs among patients instead the study relied on information provided by the parents/legal guardians. Second, the sensitivity of blood culture is known to be less than 100%. Therefore the use of a single culture bottle may have contributed to the low detection of bacteria pathogens [44]. Third, difficulty in venous sampling from young patients might have added to the low yield of bacterial pathogens. Lastly, the study could not provide sufficient bacterial isolates for in-depth characterisation of antimicrobial susceptibility pattern.

## Conclusion

In conclusion, bacteraemia was uncommon. This study highlights the need for rational use of antimicrobial prescription in febrile paediatric outpatients presenting at healthcare facilities in rural Tanzania. Accurate diagnosis of bacterial infections in Tanzania remains a challenge because of non-specific symptoms and the lack of reliable diagnostic tools. Clinicians are therefore advised to withhold prescription of antibiotics to non-severe cases of febrile illnesses unless proven by laboratory results.
